# Crown Ether-Functionalized Polyethersulfone Membranes with Potential Applications in Hemodialysis

**DOI:** 10.3390/polym17162184

**Published:** 2025-08-09

**Authors:** Madalina Oprea, Andreea Madalina Pandele, Catalin Ionel Enachescu, Iulian Vasile Antoniac, Stefan Ioan Voicu, Anca Maria Fratila

**Affiliations:** 1 Advanced Polymers Materials Group, National University of Science and Technology POLITEHNICA Bucharest, 1-7 Gheorghe Polizu, 011061 Bucharest, Romania; madalinna_09@yahoo.com (M.O.); pandele.m.a@gmail.com (A.M.P.); 2 Department of Analytical Chemistry and Environmental Engineering, Faculty of Chemical Engineering and Biotechnologies, National University of Science and Technology POLITEHNICA Bucharest, 1-7 Gheorghe Polizu, 011061 Bucharest, Romania; 3 Department of Dermatology, Elias Emergency University Hospital, 17 Bulevardul Marasti, 011461 Bucharest, Romania; catalin_enachescu@yahoo.com; 4 Faculty of Materials Science and Engineering, National University of Science and Technology POLITEHNICA Bucharest, 313 Splaiul Independentei, 060042 Bucharest, Romania; 5 Academy of Romanian Scientists, 54 Splaiul Independentei Street, District 5, 050094 Bucharest, Romania; 6 Department of Dental Medicine and Nursing, Faculty of Medicine, Lucian Blaga University of Sibiu, 550169 Sibiu, Romania; anca.fratila@ulbsibiu.ro; 7 Military Clinical Emergency Hospital of Sibiu, 550024 Sibiu, Romania

**Keywords:** crown ether, polyethersulfone, hemodialysis

## Abstract

Polyethersulfone (PES) is one of the most used synthetic polymers for the production of hemodialysis membranes, due to its appropriate features, such as biocompatibility, high permeability for low-molecular-weight proteins, high endotoxin retention ability, and resistance to sterilization processes. However, there is room for improvement regarding their anticoagulant properties when coming into contact with blood. In the present study, commercial PES membranes were plasma-treated and then chemically modified with crown ether, an organic compound that could interfere with the coagulation cascade by complexating Ca^2+^ in the blood. The physico-chemical and morphological characteristics of the membranes were determined by FT-IR, XPS, TGA, SEM, and CT analyses, while their efficiency in retaining calcium ions was evaluated via ICP-MS. The results revealed that plasma treatment with a mixture of argon and ammonia was the most effective in generating nitrogen-containing surface functional groups and that these moieties can be successfully used for the covalent functionalization of the membranes. Also, the Ca^2+^ retention ability of the PES membranes was improved by up to 30% after chemical modification with 4′-aminobenzo-15-crown-5 ether.

## 1. Introduction

Hemodialysis (HD) is a medical procedure employed to replace the renal function of patients suffering from end-stage renal disease (ESRD). Through this procedure, uremic toxins, salts, and excess fluids are filtered from the patients’ blood using a semipermeable membrane [1,2]. Hemodialysis membranes can be natural (cellulose-based) or synthetic (e.g., polysulfone-, polycarbonate-, or polyamide-based); however, studies showed that cellulose-based membranes are less hemocompatible compared to synthetic ones due to the hydroxyl groups on the polymers’ surface that can initiate the activation of the complement system, thus triggering antigen–antibody reactions [3]. The biocompatibility of synthetic membranes is associated with their hydrophobicity and increased protein adsorption ability [4], but protein adsorption is also related to membrane fouling and accelerated blood coagulation; therefore, anticoagulant strategies need to be applied in order to obtain appropriate membranes for clinical applications [5,6].

Amongst synthetic polymers used for the production of hemodialysis membranes, polysulfone (PSU) stands out in virtue of its intrinsic biocompatibility, high permeability for low-molecular-weight proteins, high endotoxin retention ability, and resistance to sterilization processes [7,8]. Polyethersulfone (PES) membranes particularly gained increasing popularity for hemodialysis applications as their chemical composition is bisphenol-A-free, compared to polysulfone, and they were shown to remove medium-sized molecules with minimal albumin loss during HD treatment [5,9]. Still, the hydrophobicity of PES membranes makes them very susceptible to biofouling; therefore, the improvement in their hemocompatibility represents a global concern that needs to be addressed [10,11]. Lowering protein adhesion rate by decreasing the hydrophobic character and surface roughness, and decreasing the blood coagulation time using anticoagulant agents are some of the strategies applied to improve the hemocompatibility of PES membranes [1,12].

Heparin, administered intravenously or immobilized on the membrane surface, showed good results in preventing blood coagulation during hemodialysis; however, the increased costs and potential side effects associated with heparin usage motivated researchers to develop novel modification techniques to obtain improved membranes with superior performance [12,13]. Surface modification (e.g., polymer blending, membrane coating, plasma treatment, physical adsorption, etc.) and the synthesis of composite membranes are amongst the most effective methods used to improve specific membrane characteristics, such as hydrophilicity and mechanical resistance, and to also prevent the pore blocking phenomenon [9,14]. A trend in developing novel hemodialysis membranes consists of the incorporation of organic or inorganic fillers in the polymeric matrix [15], such as microcrystalline cellulose (MCC) [16], magnetic nanoparticles [17], multiwalled carbon nanotubes (MWCNTs) [9,18], graphene oxide (GO) [19,20], and mixtures of GO with TiO_2_ [21] or activated carbon/ZSM-5 zeolite [22], thus resulting in composite membranes with an enhanced dialysis clearance ratio and hemocompatibility.

Surface modification is also an attractive method to decrease the hydrophobicity of PES membranes while keeping the membrane backbone unmodified [23,24]. Polysulfone has a low surface reactivity, and the introduction of functional groups on its surface is mandatory for immobilizing other chemical species [25]. Some studies have focused on blending polysulfone with other polymers such as amino-silanized poly(methyl metacrylate) (N-PMMA) [18] or co-polymers of N-vinylpyrrolidone and N-butylmethacrylate (SlipSkin™) [26,27], thus resulting in membranes with excellent fouling resistance. Membranes with superior in vitro biocompatibility, low platelet adhesion, and high toxin clearance rate were also obtained by non-solvent-induced phase separation (NIPS) of block polysulfone/poly(ethylene glycol) copolymers or via electrospinning core–shell composite fibers of PES/polyvinylpirrolidone (PVP)—Beta zeolite [28]. Likewise, a novel generation of heparin-mimicking composite membranes was developed by Wang et al. by incorporating polyacrylic acid and 2-poly-2-acrylamido-2-methylpropanesulfonic acid segments into PES solutions via in situ crosslinking polymerization using polyurethane macromers as crosslinkers [12]. Other studies consisted of the activation of PES membranes’ surface by immersion in a concentrated solution of H_2_SO_4_ for 4 h. The resulting nucleophilic SO_3_H groups were used to immobilize a nontoxic polyanionic polymer, tripolyphosphate-crosslinked chitosan (TPP-CS), to provide hydrophilic properties to the membrane surface [29]. Similarly, Saadati et al. used a self-assembly dip-coating technique to modify the surface of PES membranes with zwitterionic copolymers and reported that the zwitterion moiety significantly decreased fibrinogen fouling [30].

These chemical methods represent facile techniques to introduce functional groups that have the ability to modify the surface chemistry and improve hemocompatibility and clogging resistance by repelling plasmatic proteins. On the other hand, the methods presented above imply the usage of reactants or solvents that may have toxic effects on cells and could lead to a decrease in the mechanical properties [31]. As an alternative to chemical methods, PES membranes can be modified by physical methods such as plasma treatment. To modify the membranes, ionized gases (e.g., Ar, CO_2_, N_2_, NH_3_, O_2_, etc.) are used to bombard the material’s surface, thus generating radicals that can serve as active sites for graft polymerization or immobilization of various chemical compounds on the membrane [5,14,32]. This type of treatment has the advantage that it does not use other chemical compounds or organic solvents, and the surface modification is homogeneous and does not affect general material properties. Previous studies showed that membrane hydrophilicity was improved by plasma treatment, as demonstrated by an increase in the water flow values, reduction in pore blockage, and better flow recovery after a light cleaning process, compared to untreated membranes [33,34].

The purpose of the present study was to develop a novel generation of hemodialysis membranes by immobilizing a crown ether on the surface of plasma-modified PES membranes to improve their anticoagulant character. Crown ethers are cyclic molecules containing several ether groups [35]. They are considered supramolecular receptors that play a crucial role in the formation of host–guest complexes due to their ability to accommodate positive metal ions, coordinated to the ring of oxygen atoms inside their central cavity [36,37]. It was found that crown ethers can be effectively used in the separation of amino acids [38], lithium isotopes [39,40], and the complexation of copper [41], lead [42,43], and lithium [44] cations. Regarding blood coagulation, calcium cations (Ca^2+^) play a vital part, actively participating in the activation of blood platelets and certain coagulation factors (e.g., FVIII, FXIII, etc.) and also influencing both the protein chain association and the mechanical flexibility of the formed structure [45,46,47,48]. Therefore, crown ether-immobilized PES membranes might have the ability to mitigate blood coagulation during hemodialysis by complexating calcium cations that come into contact with the membrane surface, thus interrupting the coagulation cascade.

## 2. Materials and Methods

Surface functionalization is one of the most effective and reliable techniques for improving membrane properties, since the strong covalent bonds formed during this process ensure the retention of the functionalization agent on the membrane surface during subsequent processing steps and ensure a long shelf life of the final product. Still, the surface functionalization of polyethersulfone is thus difficult since PES is considered an inert polymer due to the lack of reactive moieties on its backbone [15]. Therefore, a two-step approach was used in this study—an initial plasma treatment with a mixture of ionized gases (NH_3_, N_2_) and argon to generate nitrogen-containing functional groups (e.g., amine, imine, amide), followed by chemical modification with 4′-aminobenzo-15-crown-5 ether using cyanuric chloride as a linking agent. The following paragraphs describe in detail the reagents and techniques used to functionalize the surface of the PES membranes.

### 2.1. Chemicals and Reagents

Commercial Polyethersulfone membranes (diameter 47 mm, porosity 0.45 µm) were supplied by Merck, Rahway, NJ, USA; Cyanuric chloride (CN) (97%, Fluka, Seelze, Germany), Ethanol (99%, Sigma Aldrich, St Louis, MO, USA), and 4′-Aminobenzo-15-crown-5 ether (AB15C5) (97%, Sigma Aldrich, St Louis, MO, USA) were used without further purification for the modification of the membranes. The water used in all experiments was deionized water.

### 2.2. Surface Functionalization of the Membranes via Plasma Treatment

The first step of this study targeted the introduction of functional groups on the surface of PES membranes using plasma treatment (fPES). Reactive gases (NH_3_, N_2_) known for the formation of nitrogen-containing functional groups (e.g., amine, imine, amide) were used in combination with argon to increase the number of active centers and functional groups on the surface. During treatment, electrons and ions from the ionized plasma bombard the material, break chemical bonds, and create active centers or free bonds at the surface. The active centers then react with the atoms in plasma and form different functional groups. After the treatment is finalized, the free bonds are neutralized by passivation at the contact with atmospheric air.

A radiofrequency plasma generator (RF = 13.56 MHz) was used for the physical modification of the membrane surface. Each membrane was placed in the reaction chamber, with the active side facing up, towards the plasma source. The chamber was vacuumed (*p* = 10 Pa), and different mixtures of discharge gases (Ar, N_2_, NH_3_, H_2_) were purged inside it. The pressure inside the reaction chamber was monitored during the treatment, and it was observed that the values ranged between 1 and 8 × 10 Pa. After 10 min, the gas supply and the radiofrequency generator were stopped, and the chamber was vented progressively before removing the membrane. Seven different samples were obtained by varying parameters such as the mixture of discharge gases, the flow rate, and the treatment time, as it is shown in Table 1.

### 2.3. Chemical Modification of the Membrane Surface with 4′-Aminobenzo-15-Crown-5 Ether

Following characterization, it was noted that the M3 sample contained the highest amount of nitrogen (nitrogen-containing functional groups) in its structure; therefore, this sample was further subjected to chemical modification. The functionalization with 4′-aminobenzo-15-crown-5 ether was realized in two steps. First, the membranes were immersed in an ethanol solution containing excess cyanuric chloride, under magnetic stirring for 2 h at 50 °C (fPES-CN). Afterwards, the membranes were impregnated with an aqueous dispersion of AB15C5 for 2 h at 60 °C (fPES-CN-AB15C5). The samples were dried at room temperature before characterization.

**Figure 1:**
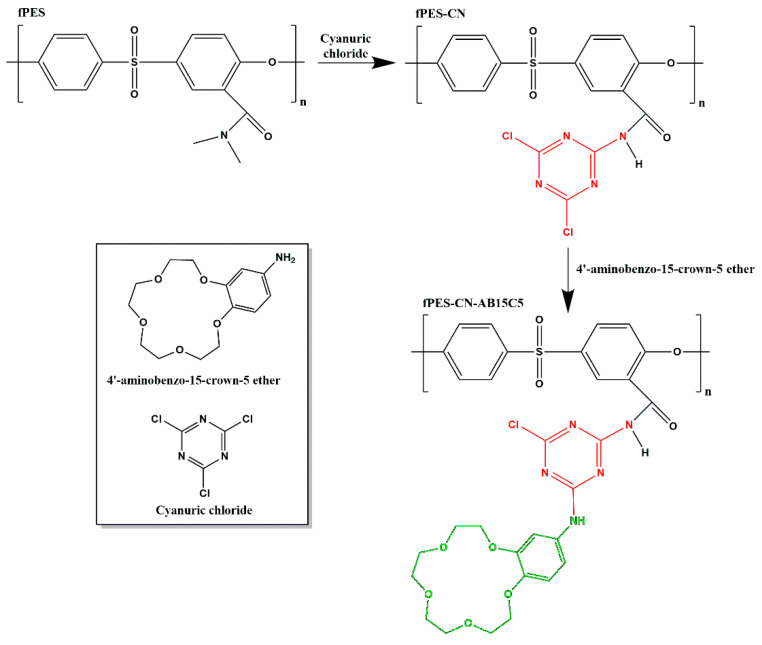
Proposed reaction mechanism for the functionalization of PES with cyanuric chloride and 4′-aminobenzo-15-crown-5 ether.

Cyanuric chloride is known to participate in nucleophilic substitution reactions, where chlorine atoms can be successively replaced by controlling the reaction temperature (once the number of substituents bound to the benzene ring increases, the reactivity decreases). In this case, cyanuric chloride acts as a connecting bridge between the amino groups on the membrane surface and the ones in the crown ether’s structure. The functionalization with 4′-aminobenzo-15-crown-5 ether was realized by a nucleophilic substitution reaction in two steps, as represented in Figure 1. In the first step, the amino groups on the membrane surface reacted with cyanuric chloride at the chlorine atom level. In the second step, the substitution was made between one of the two remaining free chlorine atoms of CN and the amino groups of AB15C5. The first reaction was conducted at 50 °C, and for the second one, the temperature was increased by 10 °C to compensate for the decreased reactivity.

### 2.4. Characterization Methods

FTIR-ATR analysis was performed using a VERTEX 70 Bruker apparatus (Bruker, Billerica, MA, USA) at an absorption interval between 600 and 4000 cm^−1^. Each membrane was scanned on both sides at different analysis points.

To record the XPS spectra, a K-alpha instrument from Thermo Fischer Scientific (Thermo Fischer Scientific, Waltham, MA, USA) with a monochromatic source of Al Ka (1486.6 eV) was used. The analysis was made at a pressure of 2 × 10^−3^ Pa.

SEM analysis was carried out using an Apreo S microscope from Thermo Fischer Scientific (Thermo Fischer Scientific, Waltham, MA, USA), equipped with a field emission electron gun with a 0.7 nm resolution. The micrographs were obtained by recording the secondary electrons resulting from an acceleration tension of 10 kV. The vacuum inside the working chamber was 1.6 × 10^2^ Pa, and the vacuum inside the column was 3.4 × 10^−4^ Pa. The analyzed samples were metalized in advance with 5 nm of gold using a magnetron-sputtering device.

The CT scans of the plasma-treated membranes were obtained using SkyScan™ 2211 from Bruker (Bruker, Billerica, MA, USA), equipped with an X-ray tube, at a tension of 30 kV and an intensity of 500 µA. The analysis was realized with a pixel dimension of 0.3 µm and a resolution of 2688 × 4032. The rotation step was fixed at 0.1 degrees, and the charge-coupled device (CCD) scanning mode was used. The three-dimensional reconstruction was realized using the NRecon software, version 1.7.1.6. The 3D images were visualized using CTVox 2.1, and Data Viewer 2.1.0 was used to observe certain bi-dimensional architectural details.

TGA analysis of the chemically modified membranes was realized using a Q500 TA Instruments device (TA Instruments, New Castle, DE, USA). The measurements were performed up to a maximum temperature of 800 °C with a heating rate of 10 °C/min in a nitrogen atmosphere.

ICP-MS was used to test the ability of the AB15C5-functionalized PES membranes to adsorb calcium ions. A calcium sulfate feed solution with a concentration of 1g/L was filtered through the membranes, and the filtrate was analyzed to determine the concentration of remaining ions using an Agilent 8800 ICP-MS Triple Quadrupole equipment (Agilent Technologies, Santa Clara, CA, USA).

## 3. Results

The physico-chemical characterization of the membranes was performed in both stages of the functionalization process. The chemical composition of the plasma-treated membranes was assessed via FT-IR and XPS, while the structural changes induced by the exposure to plasma were observed using CT. Based on this first characterization step, the membrane with optimal structure and surface functionality (M3) was selected and further functionalized with crown ether. The crown ether-functionalized membrane was characterized by FT-IR, XPS, SEM, TGA, and ICP-MS. FT-IR and XPS confirmed the presence of AB15C5 at the membrane surface, thus showing that the chemical modification was successful. SEM images and TGA curves revealed that the functionalization procedure led to an increased membrane porosity and improved thermal stability. Moreover, due to the ability of AB15C5 to complexate metal ions, an up to 30% increase in the Ca^2+^ retention efficiency was noticed in the case of the functionalized membranes following ICP-MS analysis.

### 3.1. Characterization of the Plasma-Treated PES Membranes

**Figure 2:**
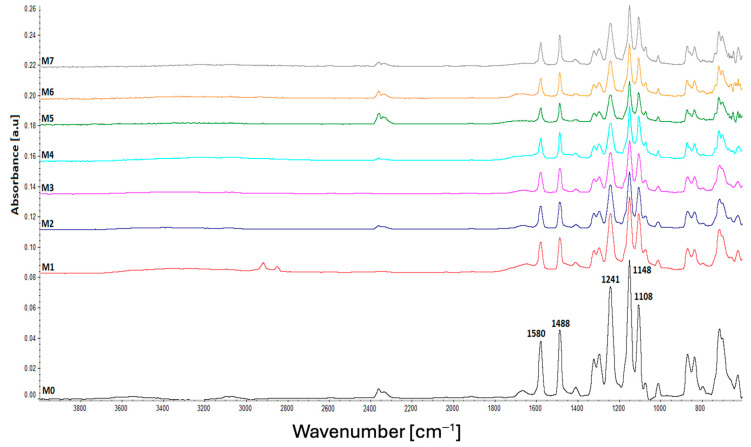
ATR FT-IR spectra of the neat and plasma-treated PES membranes.

The ATR FT-IR spectra of the neat (M0) and plasma-treated polysulfone membranes (M1-M7) are shown in Figure 2. It can be noticed that there are no substantial changes between the spectra, due to the higher sampling depth compared to the thickness of the modified layer. It is a commonly known fact that plasma treatment only generates changes in a thin, few-nanometer-sized layer on the material surface; therefore, it was expected that the FT-IR spectra would mainly show the absorption bands belonging to polyethersulfone, the new bands resulting from the modification, either not appearing at all or being of very low intensity [49].

All the samples presented the characteristic absorption bands of polyethersulfone, more specifically, the peaks at 1108 cm^−1^ and 1148 cm^−1^ attributed to the stretching vibration of the S=O bonds from the sulfone groups, the peak at 1241 cm^−1^ generated by the C-O-C bonds, and the peaks at 1488 and 1580 cm^−1^ characteristic to the C-C bonds in the aromatic phenyl and biphenyl units of PES [50,51]. The neat sample also showed two peaks at 1013 and 1074 cm^−1^, which disappeared or decreased in intensity after plasma treatment. These peaks were previously reported in the literature as corresponding to the preservatives used during the production of commercial PES membranes and are usually removed by washing the membranes with distilled water [52].

**Figure 3:**
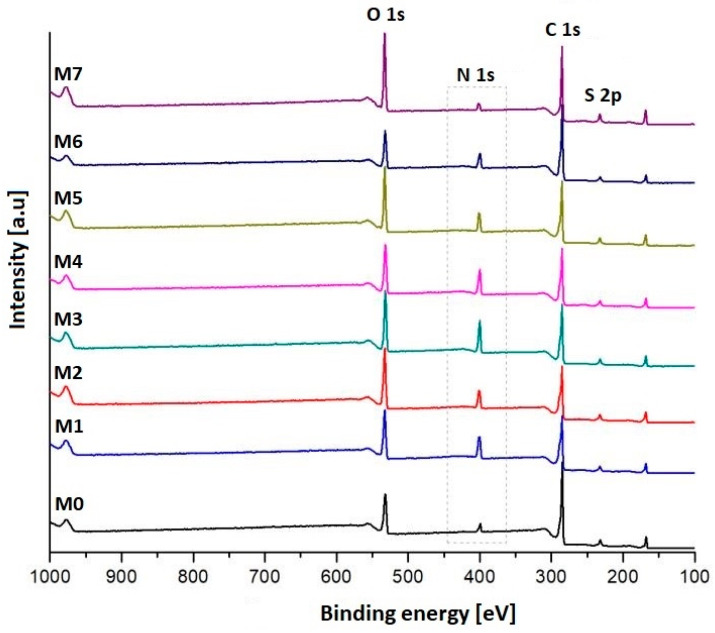
Survey XPS spectra of the neat and plasma-treated PES membranes.

According to the XPS spectrum (Figure 3), the main elements found in the untreated membrane (M0) are oxygen, carbon, and sulfur. After the plasma treatment, the peak corresponding to nitrogen was intensified, thus suggesting an increase in the percentage of nitrogen-containing functional groups in the samples. The highest N_2_ atomic percentage (~17%), was obtained for the sample treated with a mixture of discharge gases composed of Ar and NH_3_ (M3). It was also noticed that the carbon percentage of the plasma-treated samples decreased by 2.08–17.14%, while the oxygen percentage increased by 3.21–11.8%, compared to the neat sample. The changes in the sulfur content were insignificant (<2% variation) and randomly increased or decreased depending on the mixture of gases used for plasma treatment. These findings suggest that plasma treatment might cause fragmentation of the polymer chain, thus leading to a decrease in the carbon percentage while the oxygen buildup in the surface layers promotes the formation of free radicals, which take part in post-reactions after exposure to atmospheric air [53,54]. The atomic percentages previously discussed are presented in Table 2.

**Figure 4:**
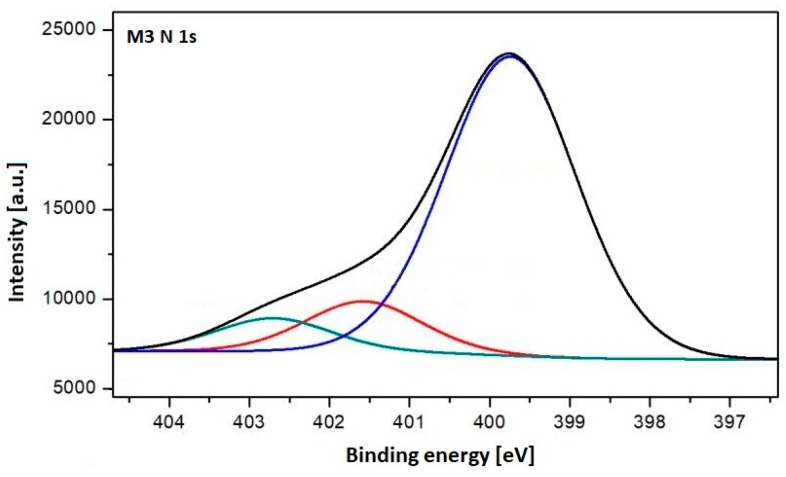
High-resolution N 1s spectrum obtained for the M3 sample—amine (blue line), amide (red line) and imine (green line).

For a better understanding of the surface chemistry, the high-resolution N 1s spectrum of the M3 sample was analyzed (Figure 4) and the data are also presented in Table 3. The spectrum revealed the presence of three peaks at approximately 399.74, 401.58, and 402.70 eV, associated with amine, amide, and imine functional groups, respectively. The results were similar to previous studies [53] and suggest that the surface of the PES membranes was functionalized with different nitrogen-containing chemical groups, particularly amine groups. In order to evaluate the substitution degree with -NH_2_ 4 groups, the amount of nitrogen 399.74 eV from the total 16.99% was measured, and we yielded a substitution degree of 13.5% [55].

**Figure 5:**
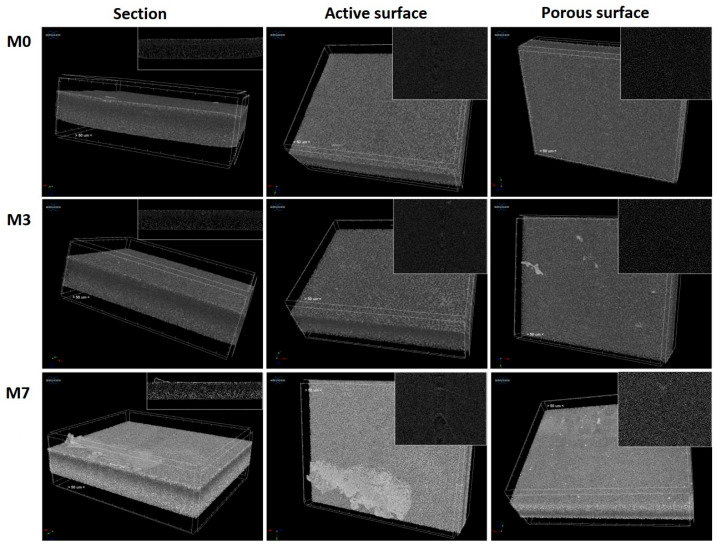
CT images obtained for the neat (M0), argon/ammonia (M3), and argon (M7) plasma-treated membranes. The scale bar represents 50 μm.

The structural changes induced by plasma treatment were further observed using CT analysis (Figure 5). For comparison purposes, the CT scans were performed on the neat (M0), argon/ammonia (M3), and argon (M7) plasma-treated membranes. All samples presented a pore architecture characteristic of asymmetric membranes, more specifically, an active surface layer and a middle layer with a high density of small-sized pores, followed by an inferior layer containing a low density of large pores [56]. After the plasma treatment, the active surface layers were thinner due to the ablation phenomenon. Moreover, compared to the neat sample (M0), the M7 sample was distinguished by a stronger luminosity both at the surface and in the depth of the membrane, thus indicating a less dense structure. Therefore, it was confirmed that plasma treatment with inert gas generated not only superficial but also bulk structural changes. The Ar/NH_3_ plasma treatment was milder, and no severe changes were noticed in the case of the M3 sample, where the active layer was still visible at the surface and the material density was similar to that of the neat membrane. Also, from CT analysis, the total porosity of the membranes was obtained using CTVox software with a value of 78 ± 3%. It was also found that the surface treatment does not affect the total porosity of the synthesized membranes.

### 3.2. Characterization of the AB15C5-Functionalized PES Membrane

**Figure 6:**
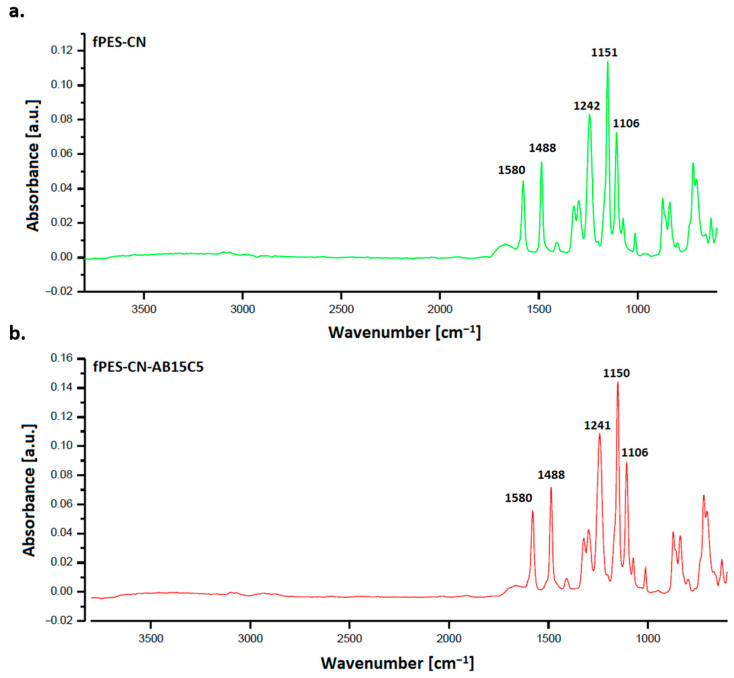
ATR FT-IR spectra of the plasma-treated PES membranes modified with cyanuric chloride (**a**) and 4′-aminobenzo-15-crown-5 ether (**b**).

The ATR FT-IR spectra of the chemically modified membranes (fPES-CN, fPES-CN-AB15C5) are shown in Figure 6. It can be observed that, after chemical modification, the spectra did not show consistent changes compared to those of the plasma-treated membrane (M3). According to previous studies, the cyanuric chloride/crown ether complex was found to generate adsorption bands at 880, 1650, 1580, and 1270 cm^−1^, due to the C=C bending, C=N stretching, N-H bending, and C-O-C stretching vibrations [57]. Therefore, the similarity between the FT-IR spectra of the plasma-treated membranes and the chemically modified ones may be related to the fact that the peaks corresponding to the newly formed bonds are shielded by the ones corresponding to the chemical bonds found in the polymer. Another cause could be due to the higher signal sampling depth compared to the size of the modified surface layer.

**Figure 7:**
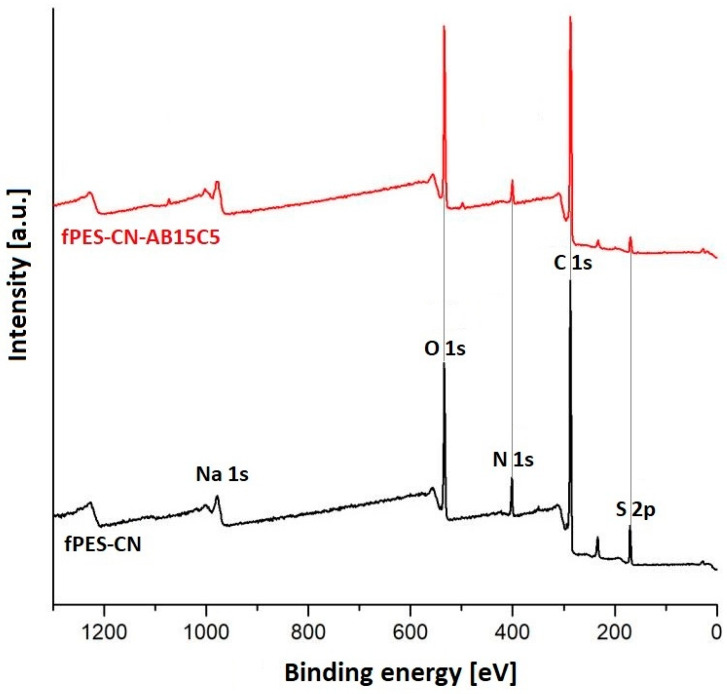
XPS spectra of the chemically modified membranes.

The survey XPS spectrum (Figure 7) indicated that, after both stages of chemical modification, the main elements present in the samples were carbon, oxygen, nitrogen, and sulfur. The small percentage of sodium found in the fPES-CN sample was probably due to the traces of water on the laboratory glassware used for the experiments. After the first functionalization stage, an increase in the carbon percentage indicated the successful attachment of cyanuric chloride to the surface of PES. The sulfur content was also increased by approximately 1%. Because no sulfur-containing compounds were used during the chemical modification procedure, it was assumed that this increase was related to the membrane swelling, pore enlargement, and partial hydrolysis of the polymer chain generated by impregnation with the ethanolic solution of cyanuric chloride [58]. These physico-chemical changes could lead to the detachment of some sulfone groups and their retention on the surface and inside the membrane pores, thus leading to a better detection of sulfur by the XPS equipment.

After the second functionalization step, it was noticed that the oxygen percentage of the fPES-CN-AB15C membrane was higher compared to fPES-CN due to the presence of the oxygen-rich crown ether molecules on the polymer surface. The sulfur percentage decreased to approximately half of the percentage in fPES-CN, thus confirming the theory explained earlier, because if indeed sulfone groups were retained on the polymer surface and in the pore volume, they were most likely washed away during the immersion of the membranes in the aqueous AB15C5 dispersion.

The nitrogen content was gradually decreased during the chemical modification stages, due to the steric hindrance effect exerted by the triazine ring of CN and macrocyclic ether structures of AB15C5 on the nitrogen-containing moieties present on the polymer surface, as it is presented in Table 4.

**Figure 8:**
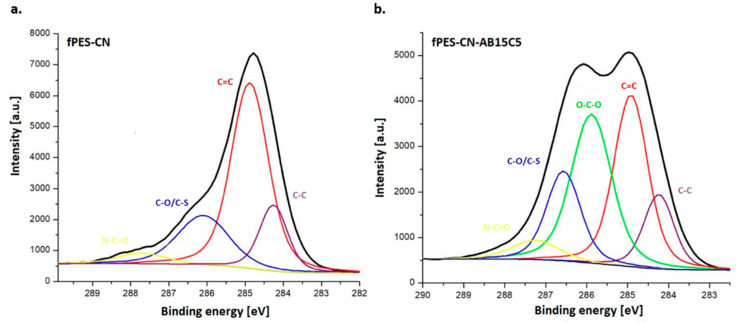
High-resolution C 1s spectra obtained for fPES-CN (**a**) and fPES-CN-AB15C5 (**b**) membranes.

For a thorough analysis of the phenomena that took place at the polymer surface after chemical modification, the high-resolution C 1s spectra of fPES-CN and fPES-CN-AB51C5 (Figure 8) were analyzed. The spectra of the fPES-CN membrane revealed the presence of four distinct peaks situated at different binding energies. Two peaks at 284.24 eV and 284.88 eV specific to C-C and C=O bonds from the aromatic phenyl and biphenyl rings, a third peak at 286.56 eV associated with C-O bonds or C-S bonds from the PES structure [59] and a fourth one at 287.27 eV corresponding to N-C=O bonds that can be found in both plasma-treated PES and in cyanuric chloride [60]. After the functionalization with crown ether, a new energy band specific to C-C bonds was present at 285.88 eV, and a slight increase in the atomic percentage specific to C-O bonds present in the crown ether could be observed, thus confirming the presence of AB15C5 at the membrane surface [61]. Also, the percent of 1.54% associated with C-Cl bonds at 200.2 eV (in the case of fPES-CN-AB15C5, with a decrease from 2.21% in the case of fPES-CN) indicates the substitution degree on the surface with crown ethers [55].

**Figure 9:**
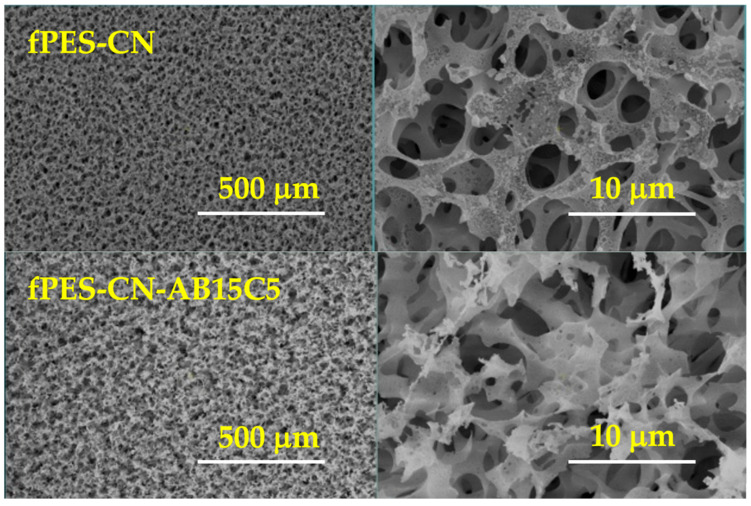
SEM images of the chemically modified PES membranes.

The SEM images in Figure 9 provided insights into the influence of the functionalization agents on the morphology of the samples. Both of the chemically modified membranes presented a slightly degraded surface and an inhomogeneous interconnected porosity with large pores. The aspect of the membranes was different after each functionalization step. As previously stated, after the analysis of the elemental atomic percentages determined by XPS, the ethanolic environment used for the initial immobilization of cyanuric chloride caused a slight degradation of the polymer and favored membrane swelling and pore enlargement. The immobilization of cyanuric chloride also generated a weak crosslinking effect, thus resulting in a rougher surface of fPES-CN compared to fPES-CN-AB1C5. Afterwards, the treatment with crown ether led to an increase in the pore diameters and a neater membrane surface; the large molecules of AB15C5 probably canceled out the weak reticulation effect of cyanuric chloride.

**Figure 10:**
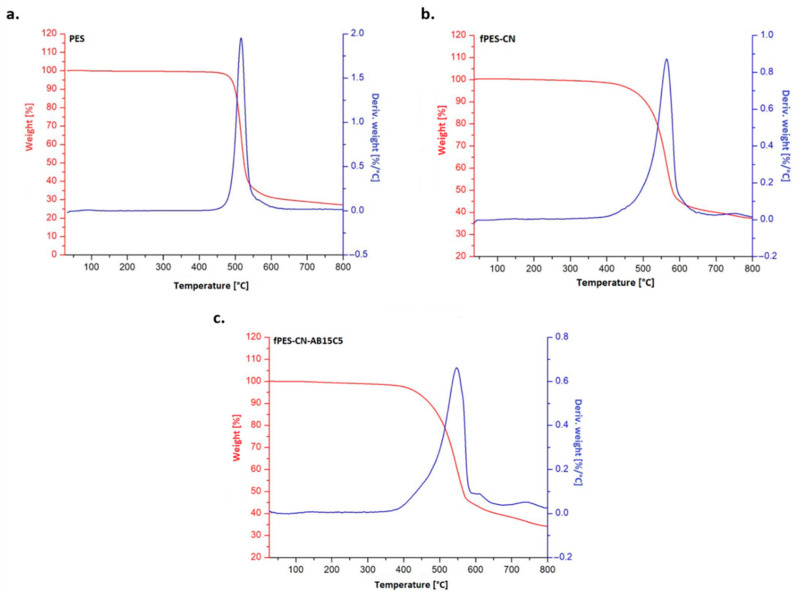
TGA and DTG curves obtained for the neat (**a**) and chemically modified PES membranes ((**b**)—fPES-CN and (**c**)—fPES-CN-AB15C5).

Thermogravimetric analysis (Figure 10) was performed in order to study the effect of the functionalization on the thermal stability of PSF. The maximum degradation temperature (Tmax), the total weight loss (WL) in the interval 25–800 °C, and the residue at 800 °C (R800) were extracted from the thermogravimetric curves and are shown in Table 5. As expected, the TGA curve obtained for the neat PES membrane (M0) showed that the material degradation occurs in a single step with a maximum degradation rate at approximately 520 °C, the recorded weight loss being attributed to the thermal degradation of the polymer backbone [62]. After functionalization with cyanuric chloride and crown ether, the main degradation peak observed in DTG was broadened and shifted towards higher values. This phenomenon confirmed the presence of the functionalization agents in the membrane structure and also suggested that the chemical modification improved the thermal stability of PSF due to the protective effect of the benzene and triazine rings in the structure of AB15C5 and CN [63]. A small peak and a shoulder were also noticed in the DTG curve of fPES-CN-AB15C5, at approximately 660 and 760 °C, and were attributed to the degradation of the nitrogen-containing groups on the membrane surface and to the carbonization of the resulting degradation products into ash.

**Figure 11:**
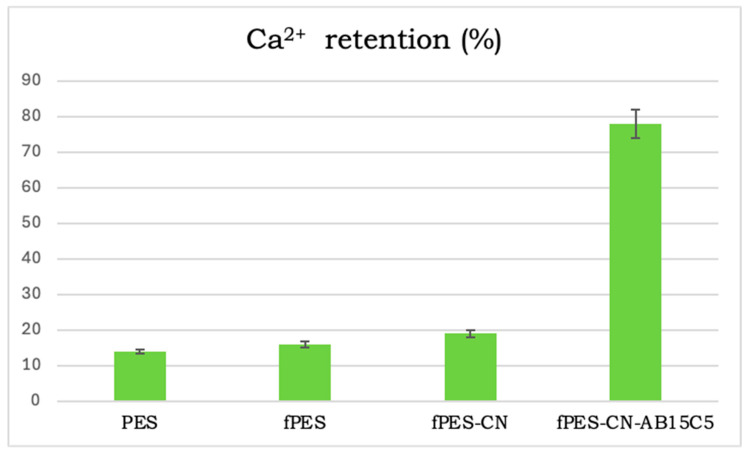
The percentages of calcium ions retained by the neat, plasma-treated, and chemically modified PES membranes.

The metal ion retention ability of the neat and functionalized membranes was tested using calcium sulfate synthetic feed solution at a physiological concentration (10 mg/dL concentration) [64] in simulated body fluid (SBF) prepared after the Kokubo method [65,66,67]. The results obtained following ICP-MS analysis are schematically represented in Figure 11. The neat PSF membrane showed a 14% adsorption efficiency of calcium ions, most likely due to the retention of the metal ions in the membrane pores. The amine, amide, and imine functional groups introduced after plasma functionalization slightly increased the Ca^2+^ retention ability due to the supplementary interactions between the metal ions and the lone pairs of electrons present in the nitrogen molecules [63]. After the first chemical functionalization step, another small increase was observed due to a mechanism similar to the one described earlier, because CN also contains nitrogen molecules in its structure. The proven ability of AB15C5 to complexate metal ions led to an up to 60% increase in Ca^2+^ retention efficiency compared to the other analyzed membranes. The result obtained from ICP-MS analysis was another confirmation of the successful chemical modification of the PSF membranes and also showed that the synthesized materials are appropriate for the desired application (mitigation of blood coagulation during hemodialysis by complexation of calcium cations that come in contact with the membrane surface).

## 4. Discussion

During hemodialysis, adverse effects such as systemic inflammation, an increase in oxidative stress, and thrombogenicity can occur when blood comes into contact with the polymeric membrane used for extracorporeal filtration. In the worst case, these events can lead to coagulation in the filtration circuit, thus resulting in reduced toxin clearance and blood loss [68]. Considering the fact that many chronic hemodialysis patients present increased hemorrhage risk when systemic anticoagulant drugs are administered, the development of hemocompatible membranes with improved anticoagulant character is an essential issue in this field [69]. Currently, the main surface modification agents employed to improve the anticoagulant character of hemodialysis membranes are heparin or heparin-like compounds, anticoagulants (e.g., citrates, argatroban, hirudin, etc.), or active proteins (e.g., albumin, antithrombin III, protein C, protein S, etc.) that either inhibit coagulation or facilitate clot lysis [70,71].

Surface modification with calcium chelating agents is a relatively new technique and is based on the fact that calcium ions play vital roles in the coagulation cascade. During hemostasis, free ionized calcium acts as a platelet activator, influences the protein conformation of most coagulation factors, and also acts as a linking agent and cofactor in enzymatic processes [72]. In this regard, it is considered that the retention of seric calcium on the hemodialysis membrane surface could prevent blood clotting by blocking the activation of factor IX, factor X, and factor II, and alter both intrinsic and extrinsic coagulation pathways.

Only two research studies focusing on calcium-chelating agent-modified membranes for hemodialysis have been published so far. In one, Liu et al. generated carboxylate groups on the surface of polysulfone membranes by modifying them with a poly (sodium alginate-acrylic acid) hydrogel via grafting polymerization. Following characterization, it was observed that the modified membranes had up to 25% Ca^2+^ removal ratio, improved toxin clearance ability—urea (~185 mL/min), creatinine (~153 mL/min), inorganic phosphorous (~120 mL/min) and β2-microglobulin (~110 mL/min) and prolonged coagulation time in terms of activated partial thromboplastin time (~490 s vs. ~50 s in neat PSF), and thrombin time (~35 s vs. ~18 s in neat PSF) [73]. In the other study, Xiao et al. used in situ growth of citric acid sodium-modified Prussian blue nanoenzymes on the surface of polyethersulfone membranes to improve their anticoagulation and antioxidant character. The presence of sodium citrate increased the activated partial thromboplastin time from ~50 s in the case of neat PES to ~95 s in the case of PBzyme-modified PES, while the enzyme component ensured a clearance capacity of 54%, 77%, and 52% for 1,1-diphenyl-2-trinitrophenylhydrazine radicals, 2,2′-azinobis(3-ethylbenzothiazoline-6-sulfonic acid ammonium salt), and hydrogen peroxide, respectively [74].

Considering these results, it can be stated that calcium-chelating agents are promising modifying agents for hemodialysis membranes. However, an important issue that needs to be tackled when applying this technique is the loss of the membrane’s anticoagulant character due to surface saturation with calcium ions. The two studies presented above are based on carboxylate-based chelation agents for calcium ions. Indeed, carboxylate groups are excellent ligands for calcium as they provide negatively charged oxygen donors with high binding affinity for the divalent calcium ion. Still, this high binding affinity could lead to rapid membrane surface saturation with Ca^2+^ and disruption of the electrolyte balance in blood. As a solution to these potential issues, this study proposes the use of AB15C5 as a chelating agent with prolonged ion sequestration ability due to the cage-like structure that complexate Ca^2+^. Initial results were promising and showed an up to 31% increase in the calcium retention ability of the AB15C5-modified PES membranes. Also, the functionalization process did not alter the porous structure of the PES membrane; therefore, the excellent filtration and separation performances were maintained intact. The main advantage of using AB15C5 as a chelating agent is the possibility to manipulate the ability of the membrane to retain calcium ions by the functionalization degree at the surface of the membrane.

## 5. Conclusions

This study was focused on developing a novel generation of polyethersulfone-based membranes with potential applications in hemodialysis. The commercial polymeric membranes were first treated with plasma to generate reactive functional groups on their surface. Afterwards, the membranes were analyzed by FT-IR, XPS, SEM, and CT to investigate which type of plasma treatment was the most effective for the desired application. According to the FT-IR spectra, all the samples presented the characteristic absorption bands of PES, and no substantial differences were observed between them. However, the more sensitive XPS analysis revealed an increase in the sample’s nitrogen content, the nitrogen percentage being distinct depending on the mixture of gases used for plasma treatment. The mixture of gases influenced not only the elemental composition of the membrane surface but also its morphological and structural features, as shown in SEM and CT images. The membrane with the highest nitrogen content (~highest amount of nitrogen-containing functional groups) was further subjected to chemical modification with crown ether in order to provide it with anticoagulant activity. The success of the functionalization reaction was confirmed by XPS and TGA analysis. In the case of chemically modified membranes, a new energy band specific to C-C bonds and an increase in the percentage of C-O bonds were noticed in the XPS spectrum, while the main degradation peak in the DTG curve was broadened and shifted towards higher values, these phenomena confirming the presence of the functionalization agent on the membrane surface. The chemical modification did not alter the sample morphology, only slight changes being visible in the SEM images of fPES-CN-AB15C5 compared to fPES (M3). The calcium ion adsorption ability was studied using ICP-MS. The obtained results showed that chemical modification with crown ether led to an up to 60% increase in Ca^2+^ retention efficiency, compared to the other analyzed membranes.

Hemodialysis is an extremely complex medical procedure that requires both rigor regarding the properties of the material and precisely controlled parameters regarding the procedure itself. Future work that will be published will be related to biological assessments of the synthesized membranes in terms of hemotoxicity, in vitro coagulation time, or platelet adhesion. Also, our future work will study the synergistic effect between the proposed solution in this study and the use of classical anticoagulants at lower concentrations.

## Figures and Tables

**Table 1:** Different parameters used for the plasma treatment.

Sample	Discharge Gas	Flow [cm^3^/min]	RF Power [W]	Pressure [Pa]	Time [min]
M1	Ar/N_2_	10/10	20	4 × 10^2^	10
M2	Ar/N_2_	10/25	20	6 × 10^2^	10
M3	Ar/NH_3_	10/10	20	5.5 × 10^2^	10
M4	Ar/NH_3_	10/25	20	7.5 × 10^2^	10
M5	Ar/H_2_ + Ar/N_2_	10/25 + 10/25	20	8 × 10^2^	5 + 5
M6	Ar/(H_2_ + N_2_)	10/(25 + 25)	20	10^3^	10
M7	Ar	10	20	2.4 × 10^2^	10

**Table 2:** Atomic percentages of the chemical elements in the samples M0-M7 determined by XPS analysis.

Sample	Atomic Percentage [%]			
	C 1s	O 1s	S 2p	N 1s
M0	73.05	17.48	4.79	4.68
M1	57.17	25.70	5.07	12.06
M2	59.70	20.69	4.27	15.34
M3	55.91	22.69	4.40	16.99
M4	58.17	21.64	4.52	15.67
M5	59.49	24.52	4.83	11.15
M6	70.97	15.48	3.81	9.74
M7	59.45	29.28	6.47	4.79

**Table 3:** High-resolution N 1s peak assignment.

N 1s Peak	Assignment	Chemical Structure *
399.74	Amine	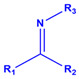
401.58	Amide	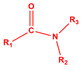
402.70	Imine	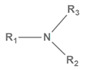

* R = H or hydrocarbon group.

**Table 4:** Atomic percentages of the chemical elements in the samples determined by XPS analysis.

Sample	Atomic Percentage [%]				
	C1s	O1s	S2p	N1s	Na1s
fPES	55.91	22.69	4.40	16.99	
fPES-CN	70.26	17.89	5.78	6.07	0.49
fPES-CN-AB15C5	70.52	21.06	2.71	4.72	-

**Table 5:** Tmax, WL, and R800 values extracted from the TGA and DTG curves.

Sample	Tmax [°C]	WL [%]	R800 [%]
PES	520	72	28
fPES-CN	565	63	37
fPES-CN-AB15C5	550	65	35

## Data Availability

Raw data are available from the corresponding author upon request.
